# Analysis of 500 anterior cruciate ligament reconstructions from a private institutional register

**DOI:** 10.1371/journal.pone.0191414

**Published:** 2018-01-19

**Authors:** Lauro Augusto Costa, Noel Oizerovici Foni, Eliane Antonioli, Rogério Teixeira de Carvalho, Isabela Dias Paião, Mário Lenza, Mário Ferretti

**Affiliations:** Hospital Israelita Albert Einstein, São Paulo, São Paulo, Brazil; Mayo Clinic Minnesota, UNITED STATES

## Abstract

**Purpose:**

The aims of this study are to describe the epidemiological characteristics of anterior cruciate ligament reconstructions in a private hospital in Brazil and to determine trends in medical practice for comparison with previous studies.

**Methods:**

We retrospectively reviewed the anterior cruciate ligament institutional register to obtain data from all patients who underwent primary anterior cruciate ligament reconstruction from July 2014 to June 2016. Descriptive statistics were used to summarize the sample. Specific statistical tests were used to assess associations between the meniscal lesion and other variables.

**Results:**

During the study period, 72.6% out of 500 patients were male. The mean age at surgery was 35.1 years. The mean age was higher among females than among males (37.3 ± 12.1 vs 34.3 ± 10.8 years). The median time from injury to surgery was 44 days. The most common femoral and tibial fixations used were suspensory fixation (60.8%) and interference screw (96%), respectively. The most commonly used graft was hamstring tendon (70.2%), followed by bone-patellar tendon-bone (28.8%). A meniscal lesion was noted in 44.8% of cases. Partial meniscectomy was performed in 69.5% of meniscal lesions, and meniscal repair was performed in 14.1% of lesions. The mean length of hospital stay was 1.4 days. The proportion of men in the group of patients with an associated meniscal lesion was higher than that in the group of patients without a meniscal lesion (p = 0.007).

**Conclusions:**

In this study, we identified that the vast majority of surgeries were performed in male patients in all age groups, and patients older than 30 years and with a short time from injury to surgery predominated. Concerning surgical technique, we noted a low rate of meniscal repair and a higher preference for the use of hamstring graft and suspensory fixation on the femoral side.

## Introduction

Anterior cruciate ligament (ACL) rupture is a common athletic injury and has been reported as the knee ligament injury that most often requires surgical reconstruction.[1] This condition has serious consequences for the injured athlete and for the general population due an increased risk of premature osteoarthritis, regardless of treatment.[2] ACL reconstruction (ACLR) has become a reliable surgical procedure to restore knee stability and prevent lesions, given that ACL deficiency can lead to cartilage and meniscal injuries.[3,4] The total number of ACLRs has been reported as between 32 and 78 procedures per 100,000 citizens/year.[1,5–9] Additionally, some studies have shown that the number of procedures performed has increased in recent years.[8,10–12]

To improve the health care system, many institutions and countries have developed ACLR registries. Such registries represent an important tool for the collection of data regarding patients and procedures and provide several benefits such as the early identification of lower clinical outcomes caused by a particular implant or surgical technique and to determine prognostic factors for the optimization of patient care.[7] Indeed, ACLR is a substantial contributor to the burden of health care costs, and costs increase further when indirect costs, such as postoperative rehabilitation and days away from employment, are included.[5] Therefore, such registries can play a role in reducing the expenses involved in the treatment of this injury, and feedback is provided from the registries to hospitals and surgeons.[5,7]

After the establishment of these registries, many studies have focused on analyzing trends in medical practice and demographic trends in ACLR. Most of these studies have shown that surgeries are performed more often in young adults and that males have a higher incidence of ACLR than females, although this difference has decreased over time.[5,7,13,14] Such studies have also shown that associated injuries are very common, such as meniscus and cartilage lesions.[6–8,13–15] Many of these studies were performed based on data obtained from healthcare systems (predominantly public) in countries with high socioeconomic status.[6,7,15] However, socioeconomic status influences the treatment that patients receive; therefore, private hospitals and developing countries can exhibit peculiar characteristics with regard to ACLR.[16] Furthermore, few epidemiological data are available regarding ACLR in countries that have no ACLR national registries; therefore, some variables have not been well described, especially for private health care systems.

The main purpose of this study is to describe the epidemiological characteristics of ACLR in a private hospital located in Brazil, a country with low socioeconomic status. We are also interested in determining trends in medical practice with a special focus on surgical technique and immediate postoperative care for comparison with previous studies.

## Materials and methods

### Prospective data collection in the ACL institutional register

The ACL register is a general database that utilizes a protocol written prospectively by a surgeon following the procedures established in the institution. The protocol comprises three sections. The first section comprises general information about patient demographics, including age, sex, body mass index (BMI), time from injury to surgery and associated meniscal lesion. The second section includes intraoperative information about ACLR. Surgery-related data, including graft type and fixation devices used, type of anesthesia, duration of the surgical procedure and use of antibiotics are reported. All other procedures performed on the injured knee, including meniscal surgery (resection or repair) are reported in the second section. The third section reports information about immediate postoperative care. This section reports on the use of postoperative antibiotic treatment, prophylactic treatment for deep venous thrombosis, the use of a postoperative drain and hospital length of stay.

### Retrospective data collection for this analysis

The study was sent to and accepted by the Ethics and Research Committee of the Hospital Israelita Albert Einstein (number 57919016.0.0000.0071). All data were analyzed anonymously so that there was no personal identification of the patients included in the registry.

A retrospective analysis of the Hospital Israelita Albert Einstein ACL registry, located in Brazil, between July 2014 and June 2016 was conducted to collect data. All patients who underwent primary ACLR in that period were included in the study. In the absence of relevant data regarding the protocol used, the patient´s medical record was analyzed to collect the information required. A single investigator obtained all information.

Patients with insufficient data and those undergoing revision ACLR and other concomitant knee ligament surgeries were excluded from this study. By these criteria, 45 cases were excluded (36 cases were male patients and 9 were female ones).

### Statistical analysis

Descriptive statistics (means and standard deviations or medians and interquartile ranges, minimum-maximum values, and absolute and relative frequencies) were used in the data evaluation to summarize the sample. The association between meniscal injury and sex was assessed using the Chi-square test. Depending on the observed distribution, the Student´s t-test or the non-parametric Mann-Whitney test was used to evaluate the associations between age, BMI, timing of surgery and meniscal injury. The statistical analysis was performed using SPSS software. The significance level adopted was p < 0.05.

## Results

**Fig 1:**
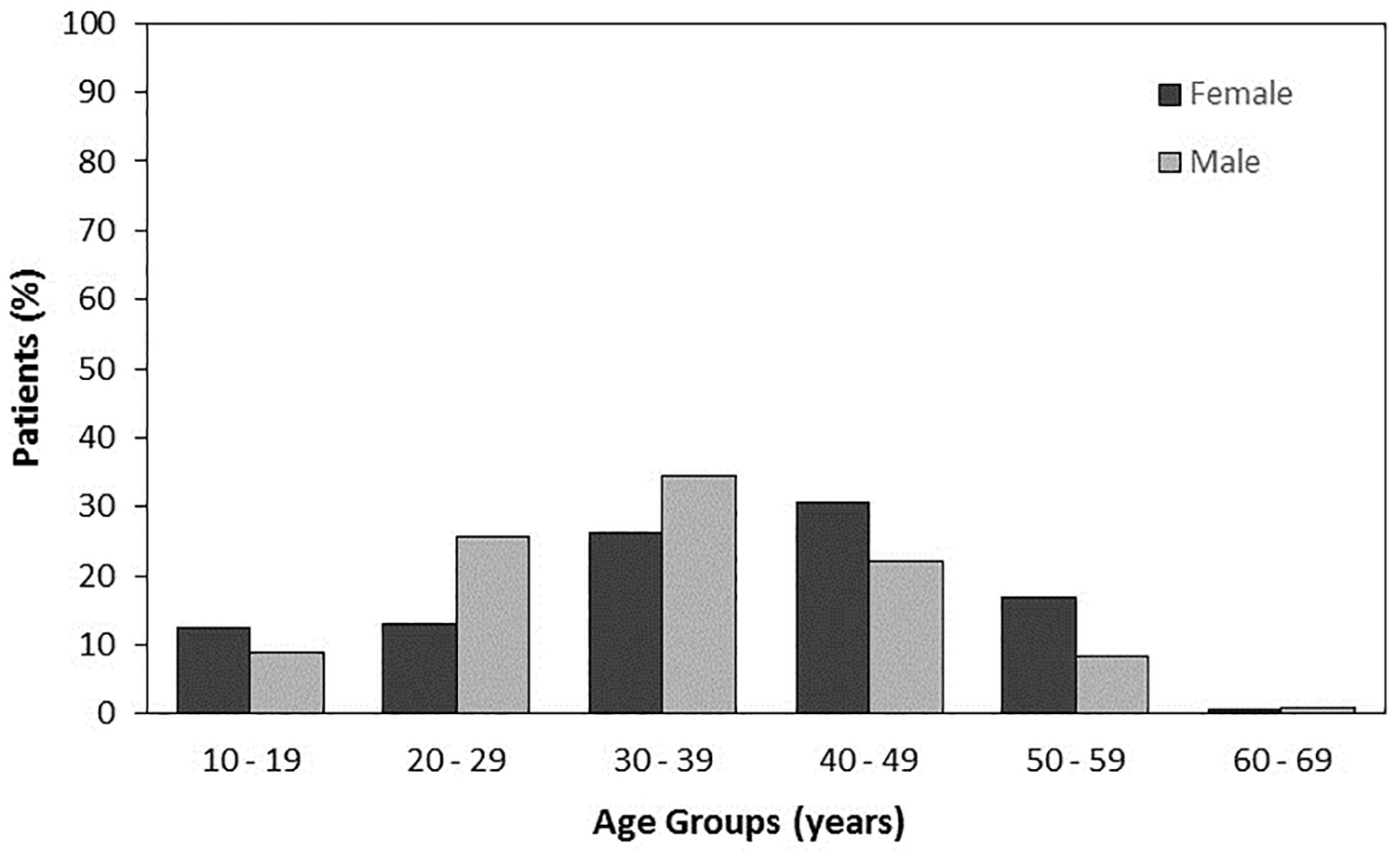
Age distribution according to sex.

A total of 500 anterior cruciate ligament primary reconstructions were compiled in the sample; 72.6% patients were male and 27.4% were female, with a mean age of 35.1 years (± 11.2 years). The patient demographics are shown in Table 1. We observed that ACLR was more prevalent in patients in the age category 30 to 39 years (32.2%). Distinct distribution depending on the sex and age of the patients were observed. ACLR was more prevalent in male patients aged from 30 to 39 years, but was more prevalent in female patients aged from 40 to 49 years (Fig 1).

**Table 1:** Patient demographics data.

Age (years)	
Mean (SD)	35.1 (11.2)
Min–max	11–67
Age (years)	n (%)
10 to 19	49 (9.8)
20 to 29	111 (22.2)
30 to 39	161 (32.2)
40 to 49	122 (24.4)
50 to 59	53 (10.6)
60 to 69	4 (0.8)
Sex	n (%)
Female	137 (27.4)
Male	363 (72.6)
Body Mass Index (BMI) (kg/m^2^)	
Mean (SD)	25.7 (3.8)
Min–max	15.6–44.1
Time from injury to surgery (days)	
Median (IIQ)	44 (21–91)
Min–max	0–2572
Number of patients	290
Meniscal lesion	n (%)
No	276 (55.2)
Yes	224 (44.8)
Side of meniscal lesion	n (%)
No	276 (55.2)
Lateral	76 (15.2)
Medial	123 (24.6)
Both menisci injured	25 (5.0)

Categorical variables are presented by absolute and relative frequencies (%)

A total of 351 (70.2%) and 144 (28.8%) reconstructions were performed using hamstring tendon (HT) and bone-patellar tendon-bone (BPTB) grafts, respectively. Quadriceps tendon graft was used in 4 (0.8%) procedures and allograft was used in just 1 (0.2%) procedure. The two most common femoral implants used were suspensory fixation (60.8%), and interference screw (34%). Interference screw was the most common graft fixation method on the tibial side (96%) as shown in Table 2.

**Table 2:** Intraoperative information.

Graft	n (%)
HT	351 (70.2)
BPTB	144 (28.8)
Quadriceps tendon	4 (0.8)
Allograft	1 (0.2)
Type of tibial fixation	n (%)
Interference screw (metal or bioabsorbable)	480 (96)
Suspensory fixation	10 (2.0)
Interference screw + secondary fixation	9 (1.8)
Staple	1 (0.2)
Type of femoral fixation	n (%)
Suspensory fixation	304 (60.8)
Interference screw (metal or bioabsorbable)	170 (34.0)
Transverse pins	24 (4.8)
Suspensory fixation + secondary fixation	2 (0.4)
Type of anesthesia	n (%)
Spinal anesthesia	433 (85.2)
General anesthesia	34 (6.8)
Others	14 (4.2)
Unknown	19 (3.8)
American Society Anesthesiology score	n (%)
I	367 (73.4)
II	106 (21.2)
Unknown	27 (5.4)
Prophylactic antibiotics	n (%)
Yes	500 (100.0)
Type of prophylactic antibiotic	n (%)
Cefazolin	385 (77.0)
Cefuroxime	86 (17.2)
Others	28 (5.6)
Unknown	1 (0.2)
Time from prophylactic antibiotic to surgery (min)	
Median (IIQ)	25 (15–30)
Min–max	-270–730
Number of patients	426
Antibiotic administration until 60 minutes prior to incision	n (%)
Yes	447 (89.4)
No	32 (6.4)
Unknown	21 (4.2)

Categorical variables are presented by absolute and relative frequencies (%); min: minutes

A meniscal lesion was reported in 224 patients (44.8%); 25 patients had both menisci injured, totaling 249 meniscal lesions; 84.3% of these were treated with some type of procedure, and the remainder were left in situ. Partial meniscectomy was the most common concomitant procedure performed (69.5%), and meniscal repair was performed in 14.1% of meniscal lesions. More isolated lesions of the medial meniscus occurred (24.6%) than isolated lesions of the lateral meniscus (15.2%); regarding the type of meniscal lesion, longitudinal lesions were the most frequent (50.6%); 68.3% of the meniscal lesions were located in the posterior third, either alone or associated with the lesions in the mid-body (Table 3).

**Table 3:** Information about meniscal lesion and type of treatment.

Meniscal lesion	n (%)
Morphology	
Longitudinal	126 (50.6)
Complex	50 (20.1)
Radial	23 (9.2)
Flap	16 (6.4)
Horizontal	10 (4.0)
Unknown	24 (9.6)
Side of lesion	
Lateral	101 (40.6)
Medial	148 (59.4)
Location	
Unknown	27 (10.8)
Anterior third	4 (1.6)
Posterior third	113 (45.4)
Body	18 (7.2)
Body and anterior third	5 (2.0)
Body and posterior third	57 (22.9)
All meniscus	25 (10)
Procedure performed	
Unknown	10 (4.0)
No	29 (11.6)
Yes	210 (84.3)
Treatment of meniscal lesion	
Unknown	10 (4.0)
Partial meniscectomy	173 (69.5)
Total meniscectomy	2 (0.8)
Meniscal repair	35 (14.1)
Left in situ	29 (11.6)

Categorical variables are presented by absolute and relative frequencies (%)

Regarding immediate postoperative care, all patients received prophylactic antibiotic treatment; more than half of the patients (56.6%) received some type of thromboembolic prophylaxis; in 25.4% of patients a drain was used after ACLR. A continuous passive motion (CPM) machine was used by almost a quarter of patients (20.2%), and cryotherapy was reported in the vast majority (70.2%). The mean hospital length of stay was 1.4 days (Table 4).

**Table 4:** Postoperative information.

Suspension of prophylactic antibiotic in 24 hours	n (%)
No	122 (24.4)
Yes	377 (75.4)
Unknown	1 (0.2)
Thromboembolic prophylaxis	n (%)
No	217 (43.4)
Yes	283 (56.6)
Thromboembolic prophylaxis	n (%)
Mechanical	232 (46.4)
Pharmacologic	12 (2.4)
Mechanical + Pharmacologic	39 (7.8)
Pharmacologic thromboembolic prophylaxis	n (%)
Enoxaparin	47 (9.4)
Rivaroxaban	4 (0.8)
Postoperative radiographic	n (%)
No	355 (71.0)
Yes	140 (28.0)
Unknown	5 (1.0)
Postoperative care	n (%)
Cryotherapy	351 (70.2)
Continuous passive motion (CPM)	101 (20.2)
Drain	n (%)
No	373 (74.6)
Yes	127 (25.4)
Time of surgery (min)	
Median (IIQ)	90 (75–110)
Min–max	40–280
Number of patients	475
Hospital length of stay (days)	
Mean (SD)	1.4 (2.1)
Median (IIQ)	1 (1–2)

Categorical variables are presented by absolute and relative frequencies (%)

Evidence of an association was found between meniscal lesion and sex (p = 0.007); the proportion of men in the group of patients with associated meniscal lesion (78.6%) was higher than that in the group of patients without meniscal lesion (67.8%). No differences were found in relation to mean age (p = 0.056), BMI (p = 0.206) and time from injury to surgery (p = 0.384) among the groups of patients with and without associated meniscal lesion (Table 5).

**Table 5:** Association between meniscal lesion, sex, age, BMI and time from injury to surgery.

		Total (n = 500)	Meniscal lesion	
			No (n = 276)	Yes (n = 224)
Sex	Female	137 (27.4%)	89 (32.2%)	48 (21.4%)
	Male	363 (72.6%)	187 (67.8%)	176 (78.6%)
			Chi-square test: p = 0.007	
Age (years)	mean (SD)	35.1 (11.2)	34.2 (10.5)	36.2 (12.0)
	min–max	11–67	11–59	12–67
			Student´s t-test: p = 0.056	
BMI (kg/m^2^)	mean (SD)	25.7 (3.8)	25.5 (3.8)	25.9 (3.9)
	min–max	15.6–44.1	18.5–40.0	15.6–44.1
			Student´s t-test: p = 0.206	
Time from injury to surgery (days)^§^	median (Q1; Q3)	44.0 (21.0; 91.0)	45.5 (23.5; 95.5)	42.0 (20.0; 87.0)
	min–max	0–2572	5–991	0–2572
	n	290	156	134
			Mann-Whitney test: p = 0.384	

Categorical variables are presented by absolute and relative frequencies (%); SD: standard deviation; Q1: first quartile; Q3: third quartile;

^§^: 210 patients excluded: insufficient data

## Discussion

This research describes trends in surgical techniques and represents an attempt to describe the epidemiology of ACLR using patient demographic data obtained from a private hospital situated in a country with low socioeconomic status. Our study focused primarily on patient characteristics, intraoperative findings and the technical aspects of the procedure. Our findings can contribute to a better understanding of the epidemiology and trends in ACLR in our geographical area.

Our study showed that the frequency of ACLR was higher in males than in females in all age groups. This finding is likely related to participation in sports, considering that males in our area play more sports that are associated with ACL injury than females. In fact, most ACL injuries occur during sporting activities; that are predominantly practiced by males.[5,7,15,17] Several studies have reported that in the general population, the incidence of ACL injury is higher in male than in female.[9,12] Although many authors report this male predominance, it appears that this sex difference is decreasing over time.[11–13,18] A study that examined United States National Center Health Statistics data showed that females accounted for 32% of the ACLRs performed in 1994 and 42% of those performed in 2004.[11] This probably reflects a growing participation in multidirectional sports by females, thus increasing their likelihood of ACL injury.

In our study, the average age of patients undergoing ACLR was higher than that found in other studies.[8,11–15,17] Interestingly, the mean age among females was even higher than that among males. Nordenvall et al. [9] reported that women are injured earlier than men, noting a maximum of incidence in patients aged 11 to 20 years. We observed that a higher proportion of individuals were older than 30 years, and almost half of females undergoing ACLR were over 40 years of age. This is consistent with the results of Mall et al.[11] which showed an increase in the number of ACLRs in patients over 40 years of age. This is probably due to the good functional results obtained after ACLR in older patients reported in recent studies and the lower risk of both revision and contralateral ACLR with increasing age.[19,20] In addition, Seng et al.[21] have suggested that older patients increasingly choose surgical treatment to remain functionally able to practice some physical activity. On the other hand, there seems to be a relationship between age and readmission after surgery. Lyman et al.[8] demonstrated that patients over 40 years old were readmitted more often than younger patients.

ACLR performed in outpatient ambulatory surgery centres has been beneficial in several aspects, including higher patient satisfaction[22] and cost reduction[23] compared to patients operated on an inpatient setting. Moreover, Lyman et al.[8] reported that rates of readmission within ninety days were significantly higher among patients who had undergone an inpatient ACLR. In the United States (US), Mall et al.[11] reported an increase from 43% of ACLRs performed in an outpatient setting in 1994 to 95% in 2006. Other authors have also reported an increase in the number of surgeries performed in this setting.[8,13,24,25] This trend also seems to be occurring in Europe. In a study describing Scandinavian ACL registries, it was noted that in Denmark, 79% of ACL reconstructions are outpatient surgeries.[26] On the other hand, all cases in our research were performed in an inpatient setting. Despite this, there were no readmissions in our study. The mean length of hospital stay was 1.4 days during the study period. Other studies that also evaluated this parameter found a similar result. Scillia et al.[23] evaluated only inpatient reconstructions in the US from 1998 to 2010 and reported that the mean length of stay was 1.6 days during the study period. The authors also identified a significant increase in total admission costs per case. Lopes et al.[27] evaluated the epidemiology of ACLR in the Brazilian public health system and reported an average length of stay of 1.8 days in 2014. In the same study, the authors showed a reduction in hospital length of stay over several years. Because of the benefits and safety of outpatient surgery, it is likely that the number of outpatient ACLR will continue to increase around the world, and Brazil will probably follow this trend.

In the current study, the rate of preference for the HT autograft was observed to be very high. Our finding is in agreement with other studies reporting a current choice for this type of graft.[6,7,15,17,28–31] In recent years, there has been a significant shift in graft choice from BPTB autograft to HT autograft.[15,25,28] To illustrate this fact, Ahlden et al.[28] and Kvist et al.[15], using a Swedish database, reported that the number of ACLRs using HT autograft has increased since 2005. In 2010, 96.1% of primary reconstructions in Sweden were performed using HT autografts.[28] Conversely, ACLR using BPTB autograft is gradually decreasing.[28] The current surgeon’s preference for HT autograft is influenced by several factors, including the ease of graft harvesting, the lower donor-site morbidity rates and less postoperative pain observed with HT autograft compared to BPTB autograft.[32] In addition, a correlation likely exists between BPTB autograft and the development of osteoarthritis, and this may influence graft selection.[33] HT autografts, in turn, have been associated with a higher risk of early revision after ACLR.[20,34] Despite this high preference rate for a specific graft type, a recent meta-analysis failed to identify which of the two types of graft is functionally better for ACLR.[32] Other sources of graft include the quadriceps tendon. A great advantage of using the quadriceps tendon is that this tendon may be used without a bone plug, resulting in minimal donor site morbidity.[35] Currently, this type of graft is less used than HT and BPTB autografts and is, therefore, also less studied.[14,31] Most orthopaedists probably do not offer this graft option when discussing surgery with the patient. However, there has been increasing interest in the use of quadriceps tendon after the report of good outcomes.[36] Regarding the prevalence of allograft indications, our results are similar to those of registry conducted in Scandinavia, where allografts have also been less indicated.[26] However, the Kaiser Permanente Anterior Cruciate Ligament Reconstruction Registry[37] and the Multicentre Orthopaedic Outcomes Network (MOON)[30], both based in the US, reported a high proportion of allograft usage (42.4% and 13%, respectively). This finding is divergent from other studies and might be explained by cultural aspects depending on geographic area that have not yet been described. In any event, the high use rate of allografts may be surprising because recent studies have reported a higher risk of early revision and poorer short-term outcomes with allografts, mainly in young patients.[14,20,34]

Several factors related to the ACL reconstruction technique, including the graft fixation method, can affect clinical outcomes. Surgeons have different options available for graft fixation, each with its own advantages and disadvantages. Therefore, graft fixation depends on graft type, surgeon preference and health insurance policy. In the present study, suspensory fixation was the most common method used on the femoral side (60.8%), followed by interference screws (34%). Transfixation pins were used in only 4% of cases. The fixation method used on the femoral side has changed with time, and this can be explained by the dissemination of the concept of anatomical ACL reconstruction. Kvist et al.[15] reported that transfixation pins were used in 60.4% of patients in 2005 but only in 5.3% of patients in 2012. Recent studies have described not only a decrease in the use of transfixation pins but also a shift from the use of transfixation pins to suspensory fixation devices on the femoral side.[25,37]

Meniscal lesions frequently accompany injuries of the ACL. This has serious implications for patients since the concomitant meniscal procedure has been associated with subsequent knee surgery.[8] In our study, meniscal injuries were identified in 44.8% of patients, a rate similar to the prevalence that has been reported by other studies.[7,12] Previous studies have reported that the incidence of concomitant meniscal injury in ACL tears ranges from 35% to 92.8%.[3,14,17,18,26,38] Medial meniscus tears have been reported as being much more prevalent than lateral meniscus tears.[4,11,12,39] Although some studies have reported on the incidence of meniscal injuries during ACL reconstruction, few studies have described the morphological pattern of the tears and their location within the meniscus. In a prospective study of 541 patients undergoing ACLR, Kluczynski et al.[4] reported that the posterior horn of both the medial and lateral meniscus was the most common location involved. Smith et al.[40] reported similar findings, with peripheral posterior horn tears of the medial meniscus being the most common type of tear in ACL-deficient knees. In accordance with the literature, our study demonstrated that most tears occur in the posterior horn and are longitudinal.

ACL injury associated with a meniscal or chondral lesion, when compared with isolated ACL injury, has been associated with an increased risk of developing osteoarthritis.[41] In view of this, predictors of intra-articular injuries associated with ACL injury have been examined in several studies.[4,39] In our study, we found an association between sex and meniscal injury, with male patients presenting a higher prevalence of meniscal injury than female patients. Similar to our study, male sex has been associated with a higher prevalence of meniscal lesion in previous studies. In a prospective study, Kluczynski et al.[4], reported an association between sex and meniscal lesion but not between meniscal lesion and other variables, such as age, BMI and time from injury to surgery. Ageberg et al.[42] also reported that female patients have less associated meniscal injury than males. Brambilla et al.[39], in a study including 988 patients, reported not only an association between meniscal lesion and male sex but also an increased risk of meniscal lesion one year after ACL injury. Unlike other studies, we found no correlations between age, BMI, time from injury to surgery and meniscal injury. Regarding time from injury to surgery, we found a median time of 44 (range 21–91) days. The timing of surgery can vary considerably between studies. Granan et al.[26] reported a relatively long time from injury to surgery based on Scandinavian registries. On the other hand, in a study enrolling ACLR at seven academic centres in the US, the median surgical time was 2.4 months.[30] Two factors may explain the differences found between the studies. First, the treatment algorithms for ACL injury may differ depending on the country. Magnussen et al.[30] reported that non-operative treatment is more common in Norway than in the US. Second, there may be longer delays in performing the surgery in some countries than in others, depending on the healthcare system adopted. The present study was performed in a private hospital, which may explain the relatively short period from injury to surgery.

Some studies have demonstrated that meniscal repair is associated with better outcomes in long-term follow-ups in osteoarthritis than partial meniscectomies.[43,44] Meniscal repairs performed with concomitant ACL reconstruction have a higher success rate than isolated meniscal repair.[44] In several studies, partial meniscectomy was reported as the preferred procedure, followed by repair for all lesions, similar to our study. [26,30,38,45–47] Nonetheless, the meniscal repair prevalence observed in this study differs from those reported in other studies. Magnussen et al.[30] reported that 39% and 22% of the meniscal lesions in patients undergoing ACL reconstruction were treated with repair in the MOON and the Norwegian Knee Ligament Registry, respectively. Previous studies in the US have reported an increasing number of meniscus repairs performed at the same time as ACL reconstructions.[48,49] This is not surprising given the increased education in the recent years regarding the chondroprotective effects of meniscus preservation.[49] Conversely, in a study conducted in Brazil, Astur et al.[50] demonstrated that less than 2% of knee surgeons in both public and private health care systems routinely perform meniscal repair at the same time as ACLR. The low rate of meniscal repair described in this study is likely due not only to the more demanding nature of the surgical technique compared to meniscectomy but also to the longer time needed for patient rehabilitation. Other possible explanations include the high cost of implants in our area. These factors may discourage some physicians from performing such procedures. Nonetheless, there have been changes in the both surgical technique and postoperative rehabilitation that may provide faster recovery, making this procedure more reliable for patients and surgeons.[51]

Considering immediate postoperative care, few studies provide information about the use of cryotherapy, CPM and postoperative intra-articular drain after ACLR. In our study, we identified that cryotherapy was used in the vast majority of patients. This is similar to the results found by Coskunsu et al.[52] and may be explained by the benefits of cryotherapy in reducing post-surgery pain in the short-term postoperative period, as reported by other studies.[53] With regard to the use of CPM, studies investigating surgeons’ preference regarding ACLR reported rates of CPM use of between 23.4% and 68.6%.[24,25,29,52] This result is surprising since several studies have shown no long-term benefits compared with standard treatment, as reported by Wright et al.[54] in a recent review. As well as CPM, the routine use of intra-articular drain after ACLR is not supported by several studies. Dhawan et al.[55] showed that the use of a drain after ACLR provides no benefit in terms of range of motion, effusion, or pain during the early postoperative period. Other studies have shown similar results.[56,57] Even so, in the current study, a quarter of the patients were fitted with a postoperative drain. These findings show that opinions are still divided among surgeons regarding several aspects of surgery.

Our study has limitations with regard to the nature of the data since it is a retrospective analysis of a database. We compare data among studies performed in different time periods, and this may influence the differences found among them. Some clinical information, such as the cause of the injury, operative details regarding chondral lesions and rehabilitation protocol, were not available. In addition, the data reflect the work of a small number of surgeons, and the observed treatment pattern might represent only the orthopaedic practices in a specific private health care system. Conversely, this study has important clinical relevance in that it provides information to the orthopaedic community that can refine our understanding of ACLR. Additionally, an ACLR registry serves to promote general improvements in the treatment of these injuries because it provides knowledge of the characteristics of patients and the current trends among surgeons.

## Conclusions

Different studies on ACLR epidemiology and medical practice present similarities and differences, that depend on the socioeconomic status of the region and the health care system adopted. In this study, which was performed at a private hospital located in a country with low socioeconomic status, we identified that the vast majority of cases involved male patients in all age groups, and patients older than 30 years and with a short time from injury to surgery predominated. Male sex was associated with a higher prevalence of meniscal lesion and all patients underwent surgery in an inpatient setting. Concerning trends in surgical technique, we noted a low rate of meniscal repair and a higher preference for the use of the HT graft and suspensory fixation on the femoral side.
